# The alternating 50 Hz magnetic field depending on the hydrophobicity of the strain affects the viability, filamentation and sensitivity to drugs of *Candida albicans*

**DOI:** 10.1371/journal.pone.0291438

**Published:** 2023-10-05

**Authors:** Dariusz Sztafrowski, Jakub Muraszko, Adam Jasiura, Patrycja Bryk, Aneta K. Urbanek, Anna Krasowska

**Affiliations:** 1 Faculty of Electrical Engineering, Wroclaw University of Science and Technology, Wroclaw, Poland; 2 Faculty of Biotechnology, University of Wroclaw, Wroclaw, Poland; 3 Faculty of Medicine, Wroclaw Medical University, Wroclaw, Poland; University of Jeddah, SAUDI ARABIA

## Abstract

In recent decades, *Candida albicans* have been the main etiological agent of life-threatening invasive infections, characterized by various mechanisms of resistance to commonly used antifungals. One of the strategies to fight *Candida* infections may be the use of an electromagnetic field. In this study, we examined the influence of the alternating magnetic field of 50 Hz on the cells of *C*. *albicans*. We checked the impact of the alternating magnetic field of 50 Hz on the viability, filamentation and sensitivity to fluconazole and amphotericin B of two, differing in hydrophobicity, strains of *C*. *albicans*, CAF2-1 and CAF 4–2. Our results indicate that using the alternating magnetic field of 50 Hz reduces the growth of *C*. *albicans*. Interestingly, it presents a stronger effect on the hydrophobic strain CAF4-2 than on the hydrophilic CAF2-1. The applied electromagnetic field also affects the permeabilization of the cell membrane. However, it does not inhibit the transformation from yeast to hyphal forms. AMF is more effective in combination with fluconazole rather than amphotericin B. Our findings confirm the hypothesis that the application of the alternating magnetic field of 50 Hz in antifungal therapy may arise as a new option to support the treatment of *Candida* infections.

## Introduction

*Candida albicans* is a polymorphic yeast-like fungus that is a part of healthy humans’ commensal gastrointestinal and genitourinary mycobiota. However, the high immunodeficiency of the host may cause opportunistic infections called candidiasis [1, 2]. The clinical spectrum of candidiasis ranges from superficial diseases such as digestive, skin, nail and genital candidiasis to systemic diseases such as blood infection (candidemia) [3]. Importantly, *C*. *albicans* can exist in three biological stages: budding yeast, pseudohyphae, and mycelium. Particularly, mycelium formation represents an important phase in the disease process. It can cause tissue damage by invading mucosal epithelial cells which next leads to candidemia [4].

Currently, candidiasis is characterized by an increase in resistance to traditional clinically used antifungal agents, such as fluconazole (FLC) or amphotericin B (AMB) [5]. The reason is several mechanisms of drug resistance developed by *C*. *albicans*, including alteration or overexpression of the drug target (mainly genes involved in ergosterol biosynthesis), upregulation of multidrug efflux transporters (principally Cdr1p belonging to ATP-binding cassette (ABC) family), activation of cellular stress responses or biofilm formation [6, 7]. Moreover, the plasticity of the mycelial form is a determining factor of drug resistance, as well as an important form during the infection phase [8]. Therefore, there is an urgent need for more precise diagnostics along with safer and more effective antifungal agents and host-directed therapies [9].

One of the strategies to combat *Candida* may be the use of an electromagnetic field. Living organisms are constantly influenced by the Earth’s magnetic field (GMF), which affects, among others, cell life cycle, DNA replication and enzyme activity [10]. Therefore, it is interesting to use the static (SMF) and alternating (AMF) magnetic fields in the research on biological processes, the values of which are several orders higher than the GMF [11]. Many studies have proven the effect of both SMF and AMF on inhibiting the growth of bacteria and fungi and slowing down their multiplication [12]. For instance, a one-day application (60 mT) has been shown to reduce the number of plaque microbes *in vitro* [13], whereas a six-hour exposure to AMF resulted in a decrease in the number of colony-forming units (CFU) compared to controls for *Staphylococcus aureus* (95.2%) and *Escherichia coli* (85%) [14]. AMF is also used in a promising cancer treatment technique called magnetic hyperthermia, where magnetic nanoparticles coated with bio-compatible molecules bound to AMF release heat [15]. Interestingly, this method was originally developed against *Staphylococcus aureus* [16].

Our previous studies have shown a positive effect of SMF on *C*. *albicans*. Cells grown in a magnetic field showed a reduced growth intensity and increased sensitivity to the FLC and AMB [17]. As there are limited data on the effects of AMF on microorganisms, especially pathogenic yeast-like fungi, the purpose of this study was to indicate if AMF influences the overall survival and permeability of *C*. *albicans* membranes, the susceptibility of *C*. *albicans* to FLC and AMB and yeast-to-hyphae transition.

## Materials and methods

### Chemicals

Chemicals and reagents were obtained from the following sources: fluconazole and amphotericin B from Sigma-Aldrich (Poznan, Poland); D-glucose and bacteriological agar from Lab Empire (Rzeszow, Poland); peptone and yeast extract (YE) from Diagmed (Warsaw, Poland); propidium iodide (PI) from BioShop (manufacturer: BioShop; distributor: Epro Science, Puck, Poland) and fetal bovine serum (FBS) from Thermo Fisher (Warsaw, Poland).

### Strains, and growth conditions

In this study two strains of *C*. *albicans* differing in hydrophobicity were used: the hydrophilic strain CAF2-1 (*ura3Δ*:*imm434/URA3*) and the hydrophobic strain CAF4-2 (*ura3Δ*::*imm434 /ura3Δ*::*imm434*). The strains were routinely grown at 28°C with agitation (120 rpm) on a YPD medium composed of 1% peptone, 1% YE and 2% glucose. To solidify the medium, agar was added (final concentration = 2%). For all experiments employing AMF, overnight pre-cultures were prepared. After incubation (YPD medium, 28°C, 120 rpm) pre-cultures were centrifuged (5 min, 2260 x g), washed with fresh YPD and subsequently resuspended in the same medium to OD_600_ = 0.1.

### Exposure of *C*. *albicans* to AMF

**Fig 1:**
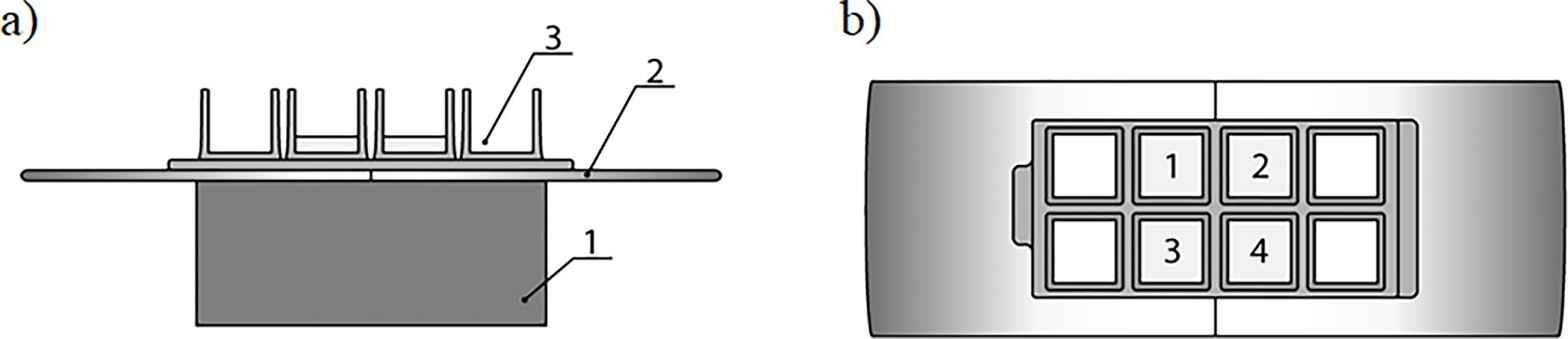
Schematic description of the test stand for exposure of *C*. *albicans* cells to AMF. a) side view: the base [1] with electromagnet [2], on which 8-well LAB-TEK chambers [3] were located; b) view from above 8-well LAB-TEK chambers located on the electromagnet [2] with simultaneous exposure of 4 biological replicates (labelled as a, b, c and d) to AMF. S and N indicate a polarity.

Exposure of *C*. *albicans* to AMF took place on a prepared stand, which consisted of the base, on which the electromagnet was placed. Prepared *C*. *albicans* cultures were applied to 8-well LAB TEK culture chambers (ThermoScientific, MA, USA) and then placed on the electromagnet. In order to maintain identical growth conditions and equal exposure to the electromagnetic field, only 4 out of 8 wells of the LAB-TEK culture chamber were used. A schematic description of the test stand is given in Fig 1. Control experiments were carried out in chambers without exposure to the electromagnetic field. Chambers were incubated for 24 hours under an alternating magnetic field (AMF) 50Hz with an induction of 35 mT. Field values were measured with BELL model 4048 meter (6120 Hanging Moss Rd. Orlando FL 32807 U.S.A).

### The viability of *C*. *albicans* in the presence of AMF

The overnight *C*. *albicans* pre-cultures were prepared as described in the 2.2 section and then applied to four centrally located wells of the LAB-TEK culture chamber. Next, the chambers with the final volume of 300 μL in each well, were incubated for 24 h at 28°C in the presence or absence of AMF. After incubation, biological material was transferred to a 96-well cell culture plate in order to measure the optical density (OD_600_) using ASYS UVM 340 (Biogenet, Józefów, Poland) microplate reader. Negative controls were wells with YPD medium alone.

### The impact of AMF on drug susceptibility of *C*. *albicans* to amphotericin B or fluconazole

*C*. *albicans* pre-cultures were prepared as described in section 2.2. Four out of eight wells of the LAB-TEK culture chamber were inoculated to a final volume of 300 μL. Subsequently, antifungal agents FLC (final conc. = 2 μg/mL) or AMB (final conc. = 0.063 μg/mL) were added to each well. These concentrations of antifungals reduced the growth of *C*. *albicans* but did not kill the cells in our previous experiments. Next, the LAB-TEK culture chambers were incubated for 24 h at 28°C with or without (control conditions) exposure to AMF. After incubation, the material was removed to a 96-well cell culture plate and the optical density (OD_600_) was measured using ASYS UVM 340 (Biogenet, Józefów, Poland) microplate reader. The viability of *C*. *albicans* was expressed in % and calculated assuming that the OD_600_ of the control conditions (section 2.4) was 100%. Negative controls were wells with YPD medium alone.

### The impact of AMF on the permeabilization of the cell membrane

The overnight *C*. *albicans* pre-cultures were prepared as described in the 2.2 section. Consistently, as presented in Fig 1, only four wells of the LAB-TEK culture chamber were inoculated to a final volume of 300 μL. Next, to each well FLC (final conc. = 2 μg/mL) or AMB (final conc. = 0.063 μg/mL) was added. Chambers were incubated at 28°C for 24h in the presence or absence of AMF. To investigate the permeabilisation of the plasma membrane (PM), propidium iodide (PI) staining was used as described before [18], with slight modifications. After incubation, *C*. *albicans* cells were stained with 6 μM PI (25°C; 5 min). Then, the cells were harvested and washed three times with phosphate-buffered saline (PBS) (5 min, 2260 *x* g). Next, microscopic slides were prepared and observed under a Zeiss Axio Imager A2 microscope (Zeiss, Oberkochen, Germany) equipped with a Zeiss Axiocam 503 mono microscope camera and a Zeiss HBO100 mercury lamp. The percentage of permeabilized cells was calculated by counting PI-positive cells out of one hundred cells in three independent repetitions for each experiment.

### Impact of AMF on yeast to yeast-to-hyphae transition

24-hour cultures of *C*. *albicans* strains in YPD medium (28°C; 120 rpm) were centrifuged (5 min, 2260 x g). The collected cells were washed with fresh YPD medium and subsequently resuspended in YPD medium to OD_600_ = 0.4. The exposure to AMF was performed in 8-well culture chambers, as described in “Exposure of *C*. *albicans* to AMF”. The hyphal transition was induced by cells suspension treatment with FBS (final conc. = 10%) for 2h at 28°C. After incubation, the samples were observed under a Zeiss Axio Imager A2 microscope equipped with a Zeiss Axiocam 503 mono microscope camera (Zeiss, Oberkochen, Germany) for the assessment of cell morphology (n = 50–100 cells in three repetitions). Zeiss ZEN 2 Blue software was used for the measurement of the length (μm) of straight hyphae.

### Statistical analysis

Each experiment was performed in triplicate. Statistical significance was determined using the Student’s t-test (Microsoft Excel software) or one-way ANOVA (α = 0.05) and Tukey’s post hoc tests (GraphPad software).

## Results

### AMF impacts *C*. *albicans* viability

**Fig 2:**
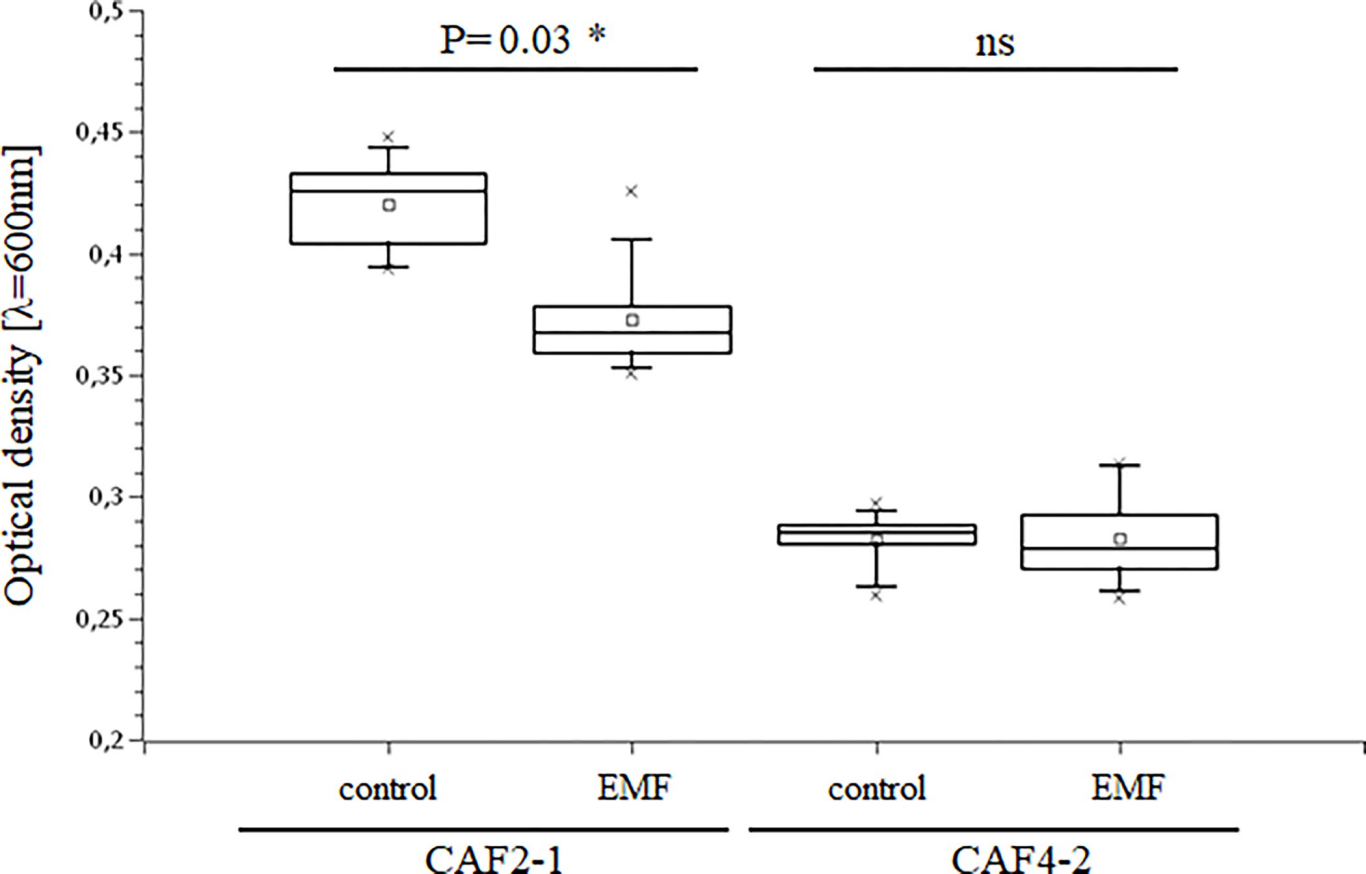
Effect of AMF on the growth of *C*. *albicans* strains. The viability expressed as optical density (OD_600_) of *C*. *albicans*’ cells exposed to AMF in comparison with cells cultured in control conditions (without AMF). Statistical analysis was performed by the t-Student test.

In the first experiment, the effect of AMF on the viability of *C*. *albicans* CAF2-1 and C. albicans CAF4-2 differing in hydrophobicity [19] was analyzed. As it turned out, both strains reached different optical density (OD_600_) values (Fig 2). Under the control conditions, the CAF2-1 strain grew much better in comparison to the CAF4-2 strain. AMF exposure showed no significant effect on the growth of the CAF4-2 strain compared to growth in control conditions. In contrast, a significant decrease in growth was observed for the hydrophilic strain, CAF2-1. The AMF reduced the growth of hydrophilic strain by around 20%.

### Drug susceptibility

Next, the synergistic impact of the AMF and antibiotics on the growth of *C*. *albicans* strains was investigated. The obtained results were compared with the viability of strains under control conditions (no antifungals, no exposure to AMF). It turned out, that generally the hydrophobic strain, CAF4-2, was more sensitive than the hydrophilic strain, CAF2-1, under the influence of both antifungals in combination with AMF. The effect of AMF on the susceptibility of the hydrophilic strain *C*. *albicans* CAF2-1 to both antifungals was insignificant. In the case of FLC, we did not observe any difference in viability (57.74% viability in control conditions and 57.78% viability after the AMF treatment), which means that the AMF did not increase the inhibitory effect of FLC. The AMB treatment showed a weak effect–the viability decreased only by 7%. In turn, the effect of both factors on the growth of the CAF4-2 strain turned out to be statistically significant. The synergistic action of AMF and FLC or AMB caused the drop of viability by 12.5% or 18.31%, respectively. Both strains used in the study were also more susceptible to AMB when exposed to the AMF. In the case of AMB influence, as in the case of FLC treatment, the hydrophobic strain was more sensitive than the hydrophilic strain. Similarly, the synergistic effect of AMB and AMF showed no statistical difference for CAF2-1. The results of the experiment are shown in Table 1.

**Table 1:** The influence of the simultaneous action of the AMF and antifungals on *C*. *albicans*.

	Viability [%]					
	No antifungals		2 μg/ml FLC		0.063 μg/ml AMB	
	Control	AMF	AMF	Control	AMF	Control
**CAF2-1**	100	79.40±2.58	57.78±6.58	57.72±3.84	75.29±6.64	82.28±9.50
**CAF4-2**	100	97.69±5.05	61.95±7.76	74.45±7.38^1^	70.69±8.58	89.00±6.74

Statistical significance was determined using the Student’s t-test

^1^—P = 0.0007***

^2^—P = 0.0004***.

### The permeabilization of the *C*. *albicans* cell membrane

**Fig 3:**
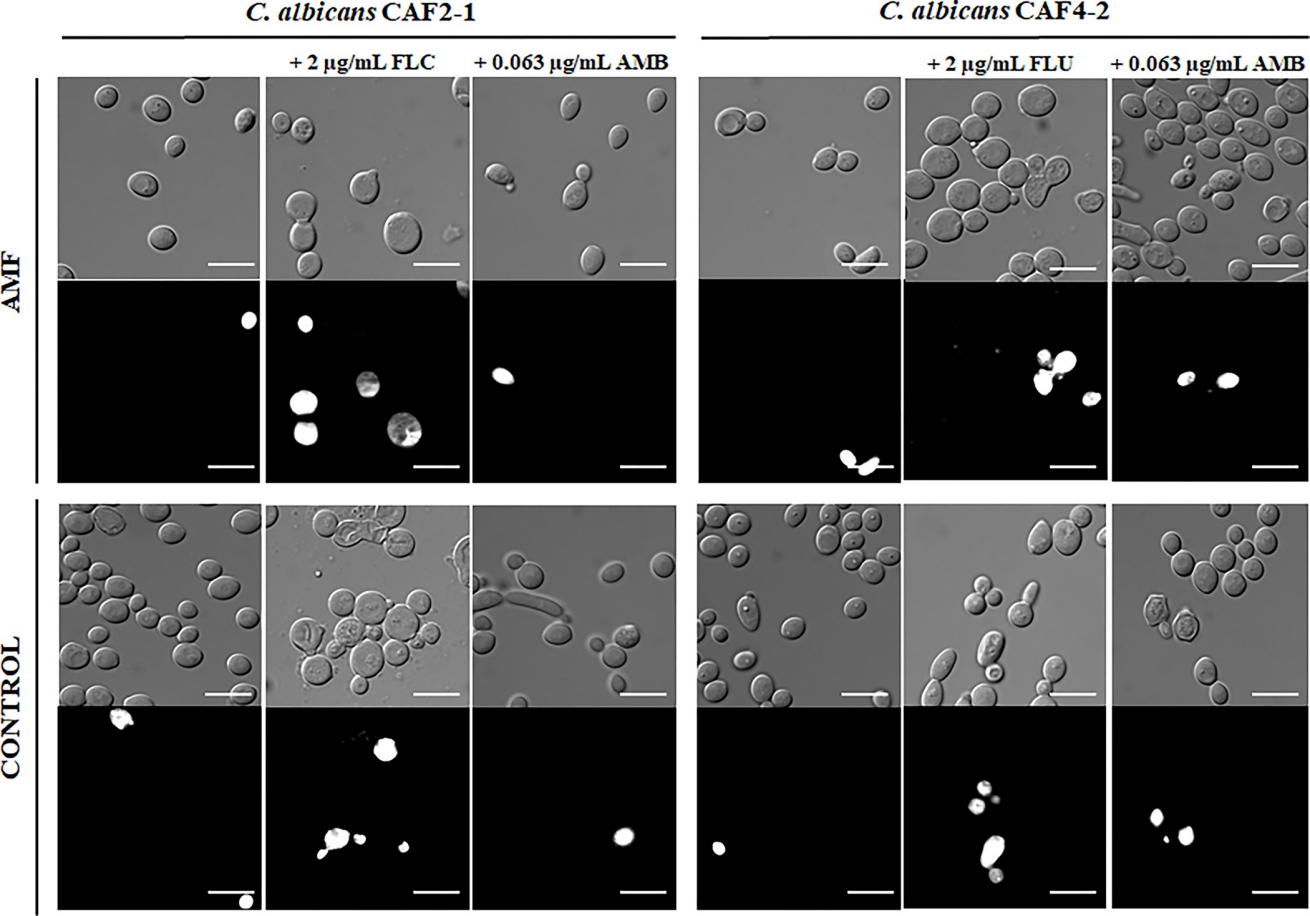
Permeabilization of *C*. *albicans* CAF2-1 and CAF4-1 strains. Representative micrographs of *C*. *albicans* CAF2-1 and CAF4-2 stained with PI after 24 h culturing in different conditions (without (control) or under exposure to AMF); scale bar = 10 μm.

The next step was to check if exposure to AMF contributes to the permeabilization of *C*. *albicans’* PM. Representative micrographs of CAF2-1 and CAF4-2 strains stained with PI are shown in Fig 3. We noticed that exposure to AMF in comparison to control conditions (without AMF) increased the amount of permeabilized cells (Table 2). However, the statistically significant result was observed only in all variants of CAF4-2, both without and with the addition of antifungals. In the case of CAF2-1 significant difference was observed only when AMF was combined with FLC. Generally, the progressive permeabilisation of PM was observed only during culturing with FLC. The addition of AMB did not change the percentage of permeabilized cells compared to the conditions without the use of this antifungal agent.

**Table 2:** Permeabilization of *C*. *albicans* strains expressed by percentage of permeabilized cells.

	Permeabilization [%]					
	No antifungals		2 μg/ml FLC		0.063 μg/ml AMB	
	AMF	Control	AMF	Control	AMF	Control
**CAF2-1**	2.19±0.79	0.70±0.10	6.13±0.24	2.19±1.28^2^	1.63±0.15	0.70±0.45^4^
**CAF4-2**	4.06±0.90	0.58±0.50^1^	4.19±1.1	0.64±0.55^3^	3.28±0.70	0.94±0.35

At least n = 100 cells per each case. The statistical analysis was performed by t-Student test

^1^—P = 0.009**

^2^—P = 0.03*

^3^—P = 0.016*

^4^—P = 0.014*.

### Yeast to yeast-to-hyphae transition

**Fig 4:**
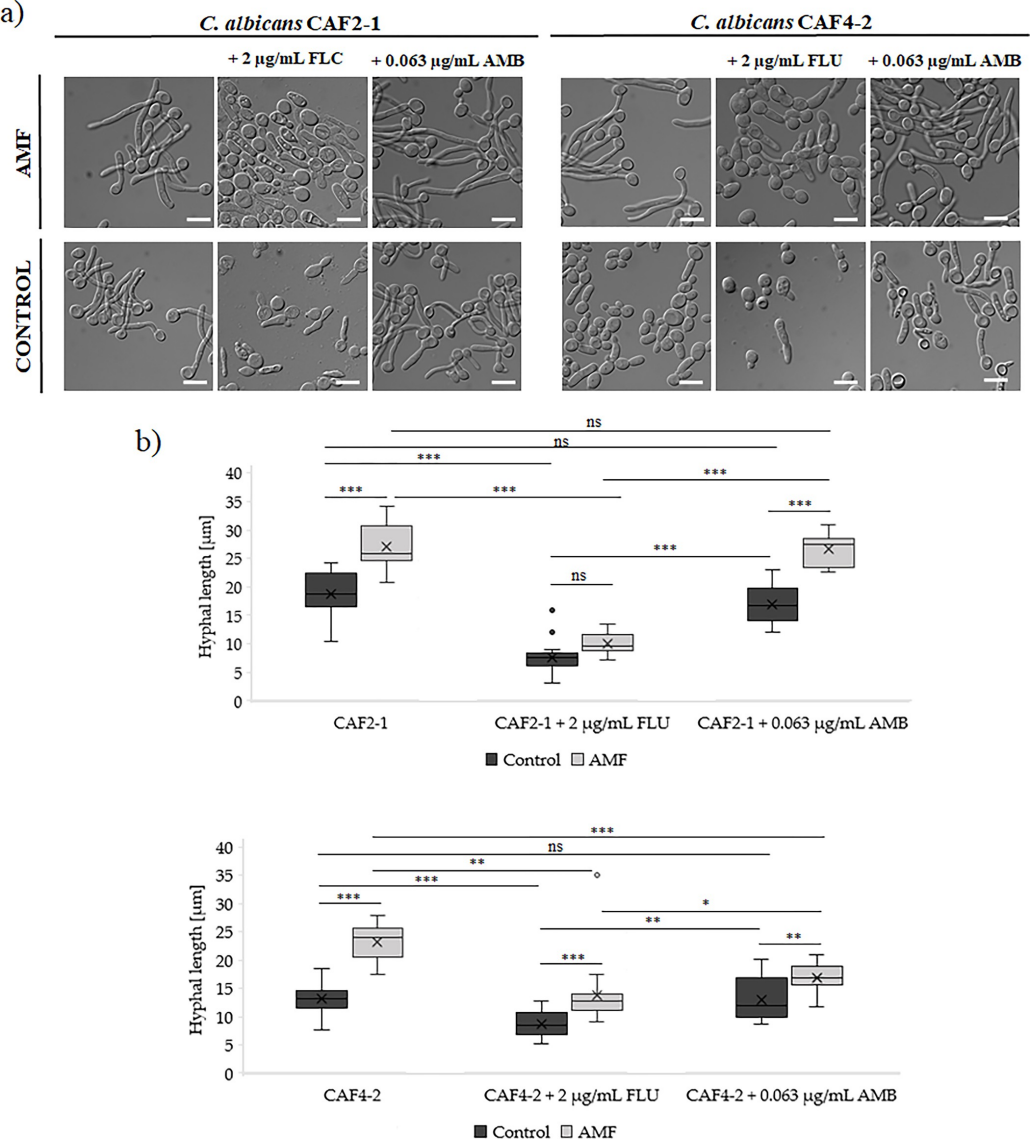
Yeast to yeast-to-hyphae transition. *C*. *albicans* cells after 2 hours incubation with FBS (28°C) with simultaneous exposure to AMF in comparison with untreated cells (control): a) microscopic images presenting cells and hyphae morphology, scale bar = 10 μm; b) hyphal length: minimal and maximal, median, Q1 and Q3. Statistical analysis was performed using one-way ANOVA (α = 0.05) and Tukey’s post hoc test (* p < 0.05; ** p < 0.01; *** p < 0.001).

The last element that we examined was the transformation of the *C*. *candida* cells into the hyphal form. The cells were incubated in the presence of FBS (10%) for 2h at 28°C. After the incubation, the slides were prepared and observed under a Zeiss Axio Imager A2 microscope. Fig 4 presents representative microscopic images (Fig 4A) and the length of hyphae (Fig 4B). It is easy to observe, that the AMF significantly induced hyphal formation in the case of both strains. The cells were elongated by about 43% in the case of CAF2-1 and 75% in the case of CAF4-2, which clearly demonstrated that exposure towards AMF does not inhibit the transition process. In variants with antifungals usage, we observed the same trend. Hyphae were also longer after combined antifungal treatment and exposure to AMF compared to control conditions (only antifungal impact). Significant inhibition of hyphal formation occurred only with the FLC administration. However, this effect was seen both for cells exposed to the combination of AMF and FLC and only under FLC influence. Thus, it can be assumed that the inhibition of hyphae formation depends more on antifungals rather than AMF. The addition of AMB had no effect on hyphal formation in the CAF2-1 strain, while the combined action of AMB and AMF on the CAF4-2 strain showed a significant effect on the shortening of hyphae length.

## Discussion

Despite the increasing amount of research on the effects of MF interactions with living organisms, many gaps remain in our knowledge. Mechanisms of electromagnetic field impact on microorganisms are not yet fully understood and due to the multitude of protocols used in biological research, there is still a contrast in literature reports on this subject [20]. Ambiguous findings are a consequence of the essence of changes occurring in microorganisms as a result of exposure to the electromagnetic field, which is the resultant of the species and the ratio of the intensity of the applied field/current to the duration of exposure [21]. Nevertheless, many studies have proven the inhibitory effect of electromagnetic fields on microorganisms. The use of MF seems particularly interesting in the case of the fungus *C*. *albicans*, which is characterized by increasing resistance to antibiotics and high mortality exceeding 60–70%. In 50% of the population, *C*. *albicans* is a part of the normal microbiota [22]. However, the overgrowth of this fungus can cause infections such as oral thrush, skin candidiasis or life-threatening invasive candidiasis, especially dangerous for immunocompromised patients [23].

One of the most important in the pathogenic life cycle of *C*. *albicans* is its ability to change morphology from yeast to hyphae (dimorphism) and thus penetration the host’s physiological barriers, damaging cells and causing invasive disease [24, 25].

In this study, we examined the influence of AMF on the cells of *C*. *albicans*. We checked if AMF affects the viability, filamentation and sensitivity to drugs of two strains of *C*. *albicans*, hydrophilic CAF2-1 and hydrophobic CAF4-2. For experiments, we selected two strains that differ in cell surface hydrophobicity (CSH) as this feature causes altered lipid metabolism of *C*. *albicans* and consequently causes different responses to treatment. Moreover, the processes of adhesion and further biofilm development depend partly on CSH [26].

Firstly, our aim was to check the effect of AMF on the viability of *C*. *albicans* strains. Comparing the growth of both strains under control conditions (without exposure to AMF), we observed a much weaker growth of the CAF4-2 strain compared to CAF2-1. This phenomenon has already been observed by us in a previous study [19]. The AMF exposure caused a significant decrease (by around 20%) in the growth of the hydrophilic strain, CAF2-1, whereas the hydrophobic strain CAF4-2 growth remained unaffected. Discussion of these results is difficult because, in the available literature, there is no data on the effect of AMF on the viability of *C*. *albicans*. However, studies on the impact of other types of MF on the growth of various microorganisms, including *C*. *albicans*, are known. For instance, there is a report of a slight decrease in viability of *C*. *albicans* CAF2-1 after 24h exposure (28°C) to SMF in comparison to the control conditions, where the average viability decreased by only 2.2% (N pole) to 3.4% (S pole) [17]. In turn, an application of a millisecond magnetic field (PEMF) resulted in a loss of pathogenic *C*. *albicans* ATCC14053 viability of up to 21% [27]. In the study of Petrini et al. (2021) the complex electromagnetic fields (CMFs) showed an antifungal effect on *C*. *albicans* colony-forming units (CFUs) as well as a decrease in *C*. *albicans* adhesion on polyacrylic resin [28]. Another study, in which *Helicobacter pylori* was undergone the low-frequency electromagnetic field (ELF-AMF), did not show any decrease in the cell viability and moreover, ELF-AMF did not affect mature biofilm. However, a decrease in the weight of biofilm formation and a decrease in the adhesive capacity of bacteria were found [29]. After the application of AMF with variable magnetic induction values of 20 mT, 40 mT, 70 mT and 90 mT, it turned out that in most cases the AMF does not significantly affect a mature biofilm of *C*. *albicans*. This finding supports the hypothesis that in most cases a mature biofilm is not significantly affected by the electromagnetic field [30]. On the other hand, stimulation of *Yarrowia lipolytica* yeast by the SMF resulted in greater increases in yeast biomass compared to the control sample [31]. In the case of *Saccharomyces cerevisiae* yeasts, the best inhibitory effect of AMF on biofilm formation was seen with a three-hour exposure to a field strength of 40 mT. Interestingly, the application of 90 mT intensity stimulated yeast cells to form biofilm [32]. All these findings prove that electromagnetic fields may either inhibit or stimulate the growth of microorganisms and biofilm formation and the results strongly depend on the electromagnetic field intensity and exposure time being used.

The next step of our research was to check the impact of AMF on the susceptibility of *C*. *albicans* to antifungals FLC and AMB, which differ in mechanisms of action. FLC belongs to the azole family of drugs and presents a common mechanism of ergosterol depletion and accumulation of sterol precursors that affect membrane structure followed by a cell wall collapse [33]. AMB, in turn, attaches to ergosterol present in the fungal cell membrane, leading to pore formation, consequent ion leakage and ultimately fungal cell death [34]. It turned out, that tested strains responded differently in the presence of AMF combined with antifungals. The hydrophilic strain *C*. *albicans* CAF2-1 was slightly more sensitive to AMB in comparison to the control conditions. In the case of FLC, any significant difference between exposure to FLC and combined FLC with AMF was observed. The synergistic action of AMF and antifungals caused more significant changes in the hydrophobic strain CAF4-2. The application of FLC and AMB in the presence of AMF caused a drop in viability by 12.5% and 18.31%, respectively. These results prove the synergistic influence of antifungals and AMF in the case of *C*. *albicans* CAF4-2 and show that the susceptibility to the synergistic treatment of AMF and drugs may depend on hydrophobicity. Generally, the hydrophobic strain is more resistant to antifungals due to overproduced ergosterol and displayed high efflux activity of the multidrug resistance Cdr1 pump [25]. However, additional exposure to AMF may disturb CSH, which results in decreased viability of this strain. Our finding is supported by the study of Sztafrowski et al. (2019), in which a statistically significant effect on *C*. *albicans* viability by combined SMF and AMB was observed. It was concluded that the synergistic effect may be due to the effect of SMF on the orientation of domains in the PM [17].

Next, we investigated the effect of AMF on the permeabilization of *C*. *albicans* PM by propidium iodide staining. It turned out that the AMF affected the membrane structure of both strains in comparison to control conditions (no antifungals, no AMF). However, the CAF4-2 strain was more sensitive than CAF2-1 and showed a higher permeabilization rate. The addition of FLC enhanced the permeabilization effect, while AMB did not cause changes compared to the control conditions where only AMF was applied. Perhaps, the reason for the lack of permeabilized cells is a relatively low concentration of AMB. In the study of Novickij et al. (2018), the influence of PMF on the permeabilization of *C*. *albicans* was investigated. Unfortunately, no detectable cell permeabilization was observed when only PMF was used. A statistically significant result was found after applying electroporation in combination with a high-power pulsed electromagnetic field (PAMF) [27].

In the end, we wanted to check the impact of AMF on hyphae formation. In a previous study, Sztafrowski et al. showed that SMF caused the shortening of *C*. *albicans* filaments. Our results showed the opposite effect. The action of AMF resulted in the elongation of the filaments (Fig 4B) [17]. This finding means that exposure of *C*. *albicans* cells to AMF, even with the addition of antifungals, stimulates cells to elongate, which clearly indicates that the electromagnetic field is a stress factor for cells.

The results obtained in this study suggest that AMF may have potential in *C*. *albicans* treatment by affecting PM viability and permeability. The use of AMF in antifungal therapy may be a new option to support the treatment of *Candida* infections. AMF has been proven to inhibit the growth of *C*. *albicans*, however, it should be noted that it had a stronger effect on the hydrophobic strain CAF4-2 than on the hydrophilic one CAF2-1. Thus, AMF-emitting devices may have great potential for concerns about antibacterial and antifungal activity. It has been shown that exposure to an electromagnetic field can induce cell modifications in shape, surface, and cytoskeleton [20], which was also evident in our research. The mechanisms of action of each AMF are not yet fully understood, which contributes to the existence of contrasts in literature, due to the multitude of approaches and protocols that can be adopted in research [35]. Consequently, investigations into methods involving electromagnetic fields to reduce *Candida*-biofilm infections are encouraged.

## Conclusions

The data obtained in the present study indicates that the hydrophobicity of *C*. *albicans* cells influences their behaviour when exposed to AMF. Both the hydrophilic and the hydrophobic strains show different patterns under the influence of AMF alone or under the synergistic effect of AMF and antifungals. In general, AMF makes cells slightly more sensitive to FLC and AMB which is manifested by reduced growth rate and increased permeabilization of membranes under the influence of AMF. Moreover, it can be concluded that AMF is a stress factor for *C*. *albicans* and may cause different effects including trigger defence mechanisms, such as the elongation of hyphae, which was observed in this study. Our findings and the state of knowledge presented above indicate the existence of potential opportunities for the practical use of the electromagnetic field in medicine. However, the impact of electromagnetic fields, including AMF, on modulating fungal physiology, development and growth must be further studied and improved for future research and application.
